# Multispectral Demosaicing Based on Iterative-Linear-Regression Model for Estimating Pseudo-Panchromatic Image

**DOI:** 10.3390/s24030760

**Published:** 2024-01-24

**Authors:** Kyeonghoon Jeong, Sanghoon Kim, Moon Gi Kang

**Affiliations:** School of Electrical and Electronic Engineering, Yonsei University, Seoul 03722, Republic of Korea; qazwxd91@yonsei.ac.kr (K.J.); shgeem@yonsei.ac.kr (S.K.)

**Keywords:** color demosaicing, color interpolation, multispectral imaging, hyperspectral imaging, pseudo-panchromatic image

## Abstract

This paper proposes a method for demosaicing raw images captured by multispectral cameras. The proposed method estimates a pseudo-panchromatic image (PPI) via an iterative-linear-regression model and utilizes the estimated PPI for multispectral demosaicing. The PPI is estimated through horizontal and vertical guided filtering, with the subsampled multispectral-filter-array-(MSFA) image and low-pass-filtered MSFA as the guide image and filtering input, respectively. The number of iterations is automatically determined according to a predetermined criterion. Spectral differences between the estimated PPI and MSFA are calculated for each channel, and each spectral difference is interpolated using directional interpolation. The weights are calculated from the estimated PPI, and each interpolated spectral difference is combined using the weighted sum. The experimental results indicate that the proposed method outperforms the State-of-the-Art methods with regard to spatial and spectral fidelity for both synthetic and real-world images.

## 1. Introduction

Commercial cameras, which capture traditional red-green-blue-(RGB) images, typically record only three colors in the visible band, and they are commonly used for general landscapes and portraits, making them one of the most popular camera types. However, with the development of various industries, there is a growing need to record or identify objects that are not easily discernible in RGB images. To meet this demand, multispectral cameras have been developed. Multispectral imaging has become an increasingly important tool in various fields, such as remote sensing [1], agriculture [2], and biomedical imaging [3]. These imaging systems capture information from multiple spectral bands, thereby providing valuable information that is not visible in traditional grayscale or RGB imaging.

**Figure 1:**
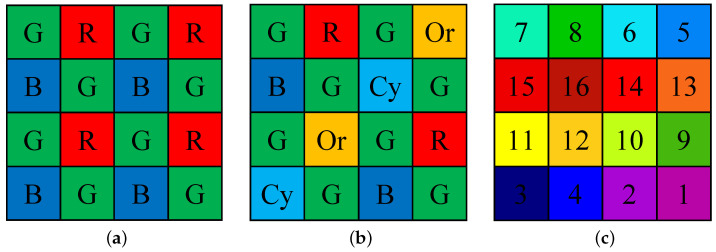
Basic CFA and MSFA patterns: (**a**) Bayer pattern [4]. (**b**) MSFA with one dominant band [5]. (**c**) MSFA with no dominant band in IMEC camera [6]. The numbers are the band numbers.

There are various methods for acquiring multispectral images, including rotating structures of different optical filters for each band. Although this approach can capture multispectral images with full resolution for each channel, it is unsuitable for capturing moving subjects. To address this issue, cameras employing the one-snapshot method are used. These cameras acquire mosaic images when a photograph is captured. The resulting mosaic image appears similar to the Bayer pattern [4] used in commercial RGB cameras, as shown in Figure 1a. The mosaic patterns of multispectral-filter arrays (MSFAs) vary depending on the manufacturer. The most commonly used pattern is a 4×4 array, which can be divided into two cases: one where there is a dominant channel, e.g., green, in Figure 1b [5], and another where all the channels have the same probability of appearance of $\frac{1}{16}$, as shown in Figure 1c [6].

A mosaic image is a two-dimensional image in which not every channel is measured at every pixel and, therefore, requires demosaicing to estimate unmeasured pixels. Since the introduction of the original snapshot camera for Bayer patterns, several demosaicing methods have been developed. There are three main traditional approaches: using the color-ratio domain, which assumes a constant ratio between colors in the local region [7]; using the color-difference domain, which assumes a constant difference between colors in the local region [8]; and using the residual domain [9] with guided filtering [10]. These methods first interpolate the dominant green channel and then interpolate the remaining R and B channels, using the interpolated G channel. Each channel is interpolated to restore high frequencies through edge estimation. Recent advances in demosaicing have led to the emergence of techniques based on deep learning, in addition to the aforementioned traditional methods. These methods typically use convolutional neural networks (CNNs) to train a network to generate an original image from a raw-image input. Gharbi et al. [11] proposed a joint-demosaicing-and-denoising method using residual networks.

Compared to Bayer filters, the MSFA is a relatively new technology. Therefore, demosaicing methods for MSFAs have been developed by modifying and advancing Bayer-pattern-based demosaicing methods. The simplest method of demosaicing MSFAs is to use weighted bilinear filters for each channel. However, this approach has the disadvantage of blurring images. To overcome this limitation, a method using the spectral-difference domain, which is similar to the color-difference domain in Bayer-pattern-based methods, was developed [12]. Additionally, the binary-tree-based-edge-sensing-(BTES) method [13] was developed, which first interpolates the centers of the unoccupied pixels. The multispectral-local-directional-interpolation-(MLDI) method [14] was also developed, which combines spectral-difference domains with BTES. However, the MLDI method was optimized for the proposed MSFA rather than a general MSFA, because the order of adjacent spectral bands must be offset to match the BTES order. Moreover, a method was developed for interpolating multispectral channels by creating a pseudo-panchromatic image (PPI) as a guide [15]. This method is suitable for any non-redundant 4×4 MSFA. In addition, a deep-learning-based multispectral-demosaicing method has been developed [16,17,18,19], which typically produces better results than traditional methods. However, deep-learning-based multispectral-demosaicing methods have a smaller dataset compared to deep-learning-based Bayer-filter-demosaicing methods. Consequently, it is not sufficient to train a complex network, and if the filter arrangement changes, the network must be retrained accordingly. In this paper, we propose a method to solve the problem that high frequencies are not accurately estimated when estimating PPI. Additionally, in conventional studies, only directional information about raw images was used in demosaicing; this is insufficient for estimating PPI. Because PPI is an image representing all channels, directional information about it must be included in the demosaicing process. Our approach builds on the following observations: (1) prior research [15] has demonstrated the usefulness of PPI for multispectral demosaicing; (2) PPI can be estimated from the high frequencies of MSFA; (3) guided filtering restores the high frequency components of a guide image while preserving details. To this end, we propose a method that uses guided filtering to estimate the PPI and then restores high frequencies for each channel by identifying edges according to the estimated PPI. Our approach is optimized for 4×4 MSFA patterns without a dominant band, but can be adapted to other patterns.

The main contributions of this study are as follows:

1. We propose a novel method for iterative-guided-filtering-pseudo-panchromatic-image-(IGFPPI) estimation that involves performing iterative guided filtering in both the horizontal and vertical directions, and combining the results.
2. The proposed guided-filtering technique is iterative and automatically determines the stopping criterion for each image.
3. We use the estimated IGFPPI to determine the weights of each channel, and we obtain the interpolated spectral-difference domain through a weighted sum of the difference between the IGFPPI and the spectral channels. Finally, we add the IGFPPI, to obtain the demosaicing result, and we follow the demosaicing order of the BTES method.

We conducted extensive experiments to compare the quantitative and qualitative results of the proposed method for the peak-signal-to-noise ratio (PSNR), the structural-similarity-index measure (SSIM) [20], and the spectral-angle-mapper-(SAM) [21] metrics to those of previously reported methods. The results indicated that the proposed method outperformed both traditional and deep-learning methods. In addition to using the synthesized data, we conducted experiments on actual images captured by IMEC cameras. The demosaicing results for these real-world images suggest that the proposed method performs well in practical situations.

The remainder of this paper is organized as follows: Section 2 presents related work. Section 3 describes the proposed method. Section 4 presents the experimental procedures and results. Section 5 presents our conclusions.

## 2. Related Work

The proposed algorithm is designed to be effective for multispectral cameras that acquire images in multispectral bands. This section presents an observational model that accurately describes the image-acquisition process using multispectral cameras. Our algorithm builds on the principles of guided image filtering and PPI estimation, which allows accurate demosaicing of multispectral images. Herein, we comprehensively review these methods.

### 2.1. Observation Model

The observation model of a multispectral camera can be expressed as follows:

(1) $$I_{k}^{c}=Q\left(\sum_{\lambda =a}^{b}E\left(\lambda \right)R{\left(\lambda \right)}_{k}T^{c}\left(\lambda \right)\right),$$

where $I_{k}^{c}$ represents the acquired pixel of channel *c* at pixel *k*; Q(·) is the quantization function; *a* and *b* represent, respectively, the spectral minimum and maximum ranges of the multispectral camera; E(λ) represents the relative spectral power distribution of the light source; $R{\left(\lambda \right)}_{k}$ is the spectral-reflectance factor of a subject at pixel *k*; and $T^{c}\left(\lambda \right)$ represents the transmittance of the MSFA channel *c*.

From the observation model, a raw image of a multispectral camera with *N* channels is defined as follows:

(2) $$I_{k}^{MSFA}=\sum_{c=1}^{N}I_{k}^{c}M_{k}^{c},$$

where $I_{k}^{MSFA}$ represents the raw image, $I_{k}^{c}$ represents the full resolution of channel *c* at pixel *k*, and $M^{c}$ represents the binary mask, which is a special type of image comprising only 0 s and 1 s that is used to represent the MSFA channel *c*.

### 2.2. Pseudo-Panchromatic Image

Mihoubi et al. proposed PPI estimation as a guide for multispectral demosaicing [15]. The PPI $I_{k}^{M}$ at pixel *k* is defined as the average image over all the channels of a multispectral image, as follows:

(3) $$I_{k}^{M}=\frac{1}{N}\sum_{c=1}^{N}I_{k}^{c}.$$

They developed a two-step algorithm for estimating the PPI. The first step is to create an initial PPI of the low-frequency components from the raw image. The initial PPI ${\bar{I}}^{M}$ is estimated using a simple Gaussian filter *M*, as follows:

(4a) $$\begin{array}{cc} {\bar{I}}^{M} & =I^{MSFA}*M, \end{array}$$

(4b) $$\begin{array}{cc} M & =\frac{1}{64}\left[\begin{array}{ccccc} 1 & 2 & 2 & 2 & 1\\ 2 & 4 & 4 & 4 & 2\\ 2 & 4 & 4 & 4 & 2\\ 2 & 4 & 4 & 4 & 2\\ 1 & 2 & 2 & 2 & 1 \end{array}\right], \end{array}$$

where $I^{MSFA}$ represents a raw image. Second, a high-frequency component is added to the initial PPI. The high-frequency component is calculated under the assumption that the local difference of the initial PPI is similar to that of the raw image, where the local difference is the difference between the value of the arbitrary pixel *k* and the weighted average value of its eight nearest neighbors $q\in {\tilde{N}}_{k}$ with the same channel. The final PPI ${\hat{I}}_{k}^{M}$ at pixel *k* is defined as follows:

(5) $${\hat{I}}_{k}^{M}=I_{k}^{MSFA}+\frac{\sum_{q\in {\tilde{N}}_{k}}\gamma_{q}\left({\bar{I}}_{q}^{M}-I_{q}^{MSFA}\right)}{\sum_{q\in {\tilde{N}}_{k}}\gamma_{q}},$$

where $\gamma_{q}$ is the weight calculated from the reciprocal of the difference between *k* and *q* in the raw image $I^{MSFA}$.

### 2.3. Guided Filtering

Guided filtering is a powerful and versatile technique for image processing that has numerous applications including denoising, deblurring, edge-preserving smoothing, and tone mapping. It is particularly useful for images with textures, where traditional filters may not preserve important features. The guided filter is a linear form and can be expressed mathematically as

(6) $$\begin{array}{c} q_{l}=a_{k}I_{l}+b_{k},\;\forall l\in \omega_{k}, \end{array}$$

where $I_{l}$ represents the guidance image, $q_{l}$ represents the filtered image, $a_{k}$ and $b_{k}$ are the filter coefficients, and *l* is the pixel coordinate in a local window $\omega_{k}$ centered at pixel *k*. For determining the filter coefficients, the cost function within the window is given as follows:

(7) $$\begin{array}{c} E\left(a_{k},b_{k}\right)=\sum_{l\in \omega_{k}}{\left(a_{k}I_{l}+b_{k}-p_{l}\right)}^{2}, \end{array}$$

and its solution is given as

(8) $$\begin{array}{cc} a_{k} & =\frac{\sum_{l\in \omega_{k}}I_{l}p_{l}-\mu_{k}{\bar{p}}_{k}}{N_{\omega }\sigma_{k}^{2}},\\ b_{k} & ={\bar{p}}_{k}-a_{k}\mu_{k}, \end{array}$$

where $\mu_{k}$, $\sigma_{k}^{2}$, and ${\bar{p}}_{k}$ represent the mean and variance of the guidance image *I* and the mean of the filtering input *p* in the local window $\omega_{k}$, respectively, and where $N_{\omega }$ represents the number of pixels in $\omega_{k}$.

## 3. Proposed Algorithm

In this section, we describe the proposed methods of the two main components. First, we explain the process of estimating the PPI from the raw image $I^{MSFA}$. Then, we describe the process of performing directional multispectral demosaicing using the estimated PPI.

### 3.1. Iterative Guided Filtering for Estimating PPI

**Figure 2:**
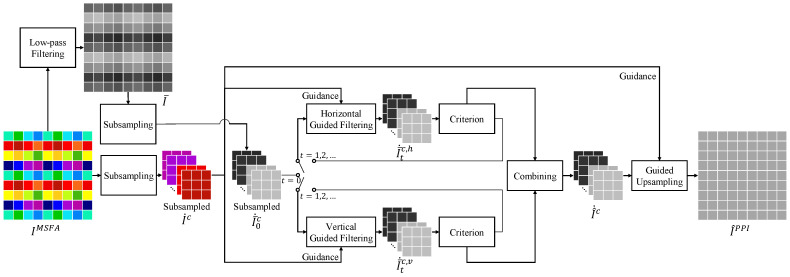
Proposed IGFPPI framework.

The proposed IGFPPI framework comprises three steps, as shown in Figure 2. First, a low-pass filter is applied to the MSFA to generate an initial image $\bar{I}$. Then, subsampling is performed, followed by iterative guided filtering. Finally, upsampling is performed to obtain the estimated PPI image.

The initial PPI, which is denoted as $\bar{I}$, includes the low-frequency components of all channels that contribute to the final PPI image. Equation (4) is used to obtain the initial estimate. Next, we perform subsampling on both $\bar{I}$ and $I^{MSFA}$ for each channel as a preprocessing step to restore the high frequencies of the final PPI. The subsampled versions of the raw image $I^{MSFA}$ and the initial PPI $\bar{I}$ in channel c are denoted as ${\dot{I}}^{c}$ and ${\dot{\bar{I}}}^{c}$, respectively. The sizes of $I^{MSFA}$ and $\bar{I}$ are (W×H), and the sizes of ${\dot{I}}^{c}$ and ${\dot{\bar{I}}}^{c}$ are $\left(\frac{W}{4}\times \frac{H}{4}\right)$, where *W* and *H* represent the width and height of the image, respectively. We use the subsampled $\dot{I^{c}}$ as the guidance image and the subsampled ${\dot{\bar{I}}}^{c}$ as the filtering input for the iterative guided filtering. Iterative guided filtering is performed separately in the horizontal and vertical directions. If the window size is increased to estimate more precise high frequencies, the estimate is closer to the MSFA image, which is the guide image. To prevent this, we calculate the horizontal and vertical directions separately, and the two results are combined to obtain the estimation. The window size used to calculate the linear coefficients is denoted as (h×v); horizontal guided filtering is used when h>v, and vertical guided filtering is used when v>h.

In the first iteration t=0, iterative guided filtering is performed in the vertical and horizontal directions, using the subsampled $\dot{I^{c}}$ as the guidance image and the subsampled ${\dot{\bar{I}}}_{0}^{c}$ as the filtering input. The equations for this process are as follows:

(9) $$\begin{array}{cc} {\dot{\bar{I}}}_{1}^{c,h}\left(i,j\right) & =a_{0}^{c,h}\left(i,j\right){\dot{I}}^{c}\left(i,j\right)+b_{0}^{c,h}\left(i,j\right),\\ {\dot{\bar{I}}}_{1}^{c,v}\left(i,j\right) & =a_{0}^{c,v}\left(i,j\right){\dot{I}}^{c}\left(i,j\right)+b_{0}^{c,v}\left(i,j\right). \end{array}$$

The pixel coordinates are represented by (i,j). For t>=1, the iterative guided filtering is repeated using the following expressions:

(10) $$\begin{array}{cc} {\dot{\bar{I}}}_{t+1}^{c,h}\left(i,j\right) & =a_{t}^{c,h}\left(i,j\right){\dot{I}}^{c}\left(i,j\right)+b_{t}^{c,h}\left(i,j\right),\\ {\dot{\bar{I}}}_{t+1}^{c,v}\left(i,j\right) & =a_{t}^{c,v}\left(i,j\right){\dot{I}}^{c}\left(i,j\right)+b_{t}^{c,v}\left(i,j\right), \end{array}$$

where $\left(a_{t}^{c,h},b_{t}^{c,h}\right)$ and $\left(a_{t}^{c,v},b_{t}^{c,v}\right)$ are the linear coefficients in the horizontal and vertical directions, respectively. The filtering inputs for iteration t+1 are the outputs of iteration *t*, i.e., ${\dot{\bar{I}}}_{t}^{c,h}$ and ${\dot{\bar{I}}}_{t}^{c,v}$, respectively.

Next, we describe the criterion block in Figure 2, which determines when the loop stops. The iterator has two conditions for stopping: (1) when each pixel stops changing, and (2) when the entire image stops changing. The loop stops when both of these conditions are satisfied.

The condition for each pixel to stop changing is determined by the following expressions:

(11) $$\begin{array}{cc} d_{t}^{c,h}\left(i,j\right) & =\left|{\dot{\bar{I}}}_{t}^{c,h}\left(i,j\right)-{\dot{\bar{I}}}_{t-1}^{c,h}\left(i,j\right)\right|,\delta_{t}^{c,h}\left(i,j\right)=\left|{\dot{\bar{I}}}_{t}^{c,h}\left(i,j-1\right)-{\dot{\bar{I}}}_{t}^{c,h}\left(i,j+1\right)\right|,\\ d_{t}^{c,v}\left(i,j\right) & =\left|{\dot{\bar{I}}}_{t}^{c,v}\left(i,j\right)-{\dot{\bar{I}}}_{t-1}^{c,v}\left(i,j\right)\right|,\delta_{t}^{c,v}\left(i,j\right)=\left|{\dot{\bar{I}}}_{t}^{c,v}\left(i-1,j\right)-{\dot{\bar{I}}}_{t}^{c,v}\left(i+1,j\right)\right|, \end{array}$$

where $d_{t}^{c,h}\left(i,j\right)$ represents the absolute difference between the results of the horizontal loops of the previous and current step, and where $d_{t}^{c,v}\left(i,j\right)$ represents the absolute difference between the results of the vertical loops of the previous and current step. These two values indicate changes in the image. As they converge to zero, there is little change in the pixels at position (i,j). Additionally, $\delta_{t}^{c,h}\left(i,j\right)$ represents the horizontal change in the result of the current step’s horizontal iteration. A value close to zero indicates that there is no change in the horizontal direction. Similarly, $\delta_{t}^{c,v}\left(i,j\right)$ represents the vertical change in the result of the current step’s vertical iteration. The criterion for pixel change is determined by multiplying these two expressions, as follows:

(12) $$\begin{array}{cc} D_{t}^{c,h}\left(i,j\right) & =d_{t}^{c,h}\left(i,j\right)\cdot \delta_{t}^{c,h}\left(i,j\right),\\ D_{t}^{c,v}\left(i,j\right) & =d_{t}^{c,v}\left(i,j\right)\cdot \delta_{t}^{c,v}\left(i,j\right). \end{array}$$

The pixel change stops when $D_{t}^{c,h}\left(i,j\right)<\epsilon_{pixel}$ for the horizontal direction and when $D_{t}^{c,v}\left(i,j\right)<\epsilon_{pixel}$ for the vertical direction, where $\epsilon_{pixel}$ represents a predefined threshold.

The global condition under which the loop stops is calculated using the following expressions:

(13) $$\begin{array}{cc} MAD^{c,h}\left(t\right) & =\frac{1}{\dot{W}\times \dot{H}}\sum_{i=1}^{\dot{H}}\sum_{j=1}^{\dot{W}}d_{t}^{c,h}\left(i,j\right),\\ MAD^{c,v}\left(t\right) & =\frac{1}{\dot{W}\times \dot{H}}\sum_{i=1}^{\dot{H}}\sum_{j=1}^{\dot{W}}d_{t}^{c,v}\left(i,j\right), \end{array}$$

where $\dot{W}$ and $\dot{H}$ represent the width and height of the subsampled image, respectively. The mean absolute difference (MAD) is a measure of the extent to which the entire image changes and is calculated as the average absolute value of the difference between the results of the previous and current steps. Ye et al. determined the convergence based solely on the MAD value [22]. However, our focus is the convergence of the difference between the current and previous MADs to zero, rather than the value of the MAD approaching zero. This is because the MAD may not converge to zero, owing to the conditions that prevent each pixel from changing. The difference in MAD between the current and previous steps is calculated as follows:

(14) $$\begin{array}{cc} \Delta_{MAD}^{c,h}\left(t\right) & =MAD^{c,h}\left(t\right)-MAD^{c,h}\left(t-1\right),\\ \Delta_{MAD}^{c,v}\left(t\right) & =MAD^{c,v}\left(t\right)-MAD^{c,v}\left(t-1\right). \end{array}$$

The final number of iterations is determined by finding the smallest value of *t* that satisfies both $\Delta_{MAD}^{c,h}\left(t\right)<\epsilon_{global}$ and $\Delta_{MAD}^{c,v}\left(t\right)<\epsilon_{global}$, which is defined as *T*.

The process of weighting and summing the results obtained by guided filtering in the vertical and horizontal directions with the number of iterations obtained earlier is as follows:

(15) $$\begin{array}{cc} {\dot{\hat{I}}}^{c}\left(i,j\right) & =\frac{w^{c,h}\left(i,j\right){\dot{\bar{I}}}_{T}^{c,h}\left(i,j\right)+w^{c,v}\left(i,j\right){\dot{\bar{I}}}_{T}^{c,v}\left(i,j\right)}{w^{c,h}\left(i,j\right)+w^{c,v}\left(i,j\right)}, \end{array}$$

where $w^{c,h}\left(i,j\right)$ and $w^{c,v}\left(i,j\right)$ are the weights in the horizontal and vertical directions, respectively, and are defined as follows:

(16) $$\begin{array}{cc} w^{c,h}\left(i,j\right) & =\frac{1}{D_{T}^{c,h}\left(i,j\right)},\\ w^{c,v}\left(i,j\right) & =\frac{1}{D_{T}^{c,v}\left(i,j\right)}, \end{array}$$

where a small criteria value contributes to a large weight.

The final step involves guided upsampling of the subsampled channel ${\dot{\hat{I}}}^{c}$ to generate the final PPI image. To achieve this, we set the window size for the linear coefficients in guided filtering to h=v, and we then upsample the image for each channel to the position of the raw image. The guided upsampling is expressed by the following equation:

(17) $$\begin{array}{cc} {\hat{I}}^{PPI}\left(4i+m,4j+n\right)=a_{T}^{c}\left(i,j\right){\dot{I}}^{c}\left(i,j\right)+b_{T}^{c}\left(i,j\right), & \\ \left(m,n\right)\in {\left[0,1,2,3\right]}^{2}, & \end{array}$$

where $\left(m,n\right)\in {\left[0,1,2,3\right]}^{2}$ determines the grid for upsampling and depends on the subsampled channel *c*. The indices (m,n) represent the position of a pixel within a 4×4 block. For example, if c=1 in Figure 1c, (m,n) is (3,3).

(18) $$\begin{array}{cc} \Delta_{s1}^{c}\left(i,j\right) & =\frac{\gamma_{s0}^{NW}\Delta_{s0}^{c}\left(i-2,j-2\right)+\gamma_{s0}^{NE}\Delta_{s0}^{c}\left(i-2,j+2\right)+\gamma_{s0}^{SE}\Delta_{s0}^{c}\left(i+2,j+2\right)+\gamma_{s0}^{SW}\Delta_{s0}^{c}\left(i+2,j-2\right)}{\gamma_{s0}^{NW}+\gamma_{s0}^{NE}+\gamma_{s0}^{SE}+\gamma_{s0}^{SW}}. \end{array}$$

(19) $$\begin{array}{c} \gamma_{s0}^{NW}=\frac{1}{2\left|{\hat{I}}^{PPI}\left(i-2,j-2\right)-{\hat{I}}^{PPI}\left(i,j\right)\right|+\left|{\hat{I}}^{PPI}\left(i-1,j-1\right)-{\hat{I}}^{PPI}\left(i+1,j+1\right)\right|},\\ \gamma_{s0}^{NE}=\frac{1}{2\left|{\hat{I}}^{PPI}\left(i-2,j+2\right)-{\hat{I}}^{PPI}\left(i,j\right)\right|+\left|{\hat{I}}^{PPI}\left(i-1,j+1\right)-{\hat{I}}^{PPI}\left(i+1,j-1\right)\right|},\\ \gamma_{s0}^{SE}=\frac{1}{2\left|{\hat{I}}^{PPI}\left(i+2,j+2\right)-{\hat{I}}^{PPI}\left(i,j\right)\right|+\left|{\hat{I}}^{PPI}\left(i+1,j+1\right)-{\hat{I}}^{PPI}\left(i-1,j-1\right)\right|},\\ \gamma_{s0}^{SW}=\frac{1}{2\left|{\hat{I}}^{PPI}\left(i+2,j-2\right)-{\hat{I}}^{PPI}\left(i,j\right)\right|+\left|{\hat{I}}^{PPI}\left(i+1,j-1\right)-{\hat{I}}^{PPI}\left(i-1,j+1\right)\right|}. \end{array}$$

(20) $$\begin{array}{c} \Delta_{s2}^{c}\left(i,j\right)=\frac{\gamma_{s1}^{N}\Delta_{s1}^{c}\left(i-2,j\right)+\gamma_{s1}^{E}\Delta_{s1}^{c}\left(i,j+2\right)+\gamma_{s1}^{S}\Delta_{s1}^{c}\left(i+2,j\right)+\gamma_{s1}^{W}\Delta_{s1}^{c}\left(i,j-2\right)}{\gamma_{s1}^{N}+\gamma_{s1}^{E}+\gamma_{s1}^{S}+\gamma_{s1}^{W}}. \end{array}$$

(21) $$\begin{array}{c} \gamma_{s1}^{N}=\frac{1}{2\left|{\hat{I}}^{PPI}\left(i-2,j\right)-{\hat{I}}^{PPI}\left(i,j\right)\right|+\left|{\hat{I}}^{PPI}\left(i-1,j\right)-{\hat{I}}^{PPI}\left(i+1,j\right)\right|},\\ \gamma_{s1}^{E}=\frac{1}{2\left|{\hat{I}}^{PPI}\left(i,j+2\right)-{\hat{I}}^{PPI}\left(i,j\right)\right|+\left|{\hat{I}}^{PPI}\left(i,j+1\right)-{\hat{I}}^{PPI}\left(i,j-1\right)\right|},\\ \gamma_{s1}^{S}=\frac{1}{2\left|{\hat{I}}^{PPI}\left(i+2,j\right)-{\hat{I}}^{PPI}\left(i,j\right)\right|+\left|{\hat{I}}^{PPI}\left(i+1,j\right)-{\hat{I}}^{PPI}\left(i-1,j\right)\right|},\\ \gamma_{s1}^{W}=\frac{1}{2\left|{\hat{I}}^{PPI}\left(i,j-2\right)-{\hat{I}}^{PPI}\left(i,j\right)\right|+\left|{\hat{I}}^{PPI}\left(i,j-1\right)-{\hat{I}}^{PPI}\left(i,j+1\right)\right|}. \end{array}$$

### 3.2. Directional Multispectral Demosaicing

**Figure 3:**
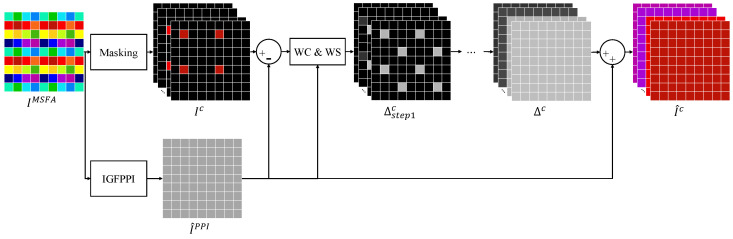
Proposed framework for directional multispectral demosaicing.

In this section, we present the proposed multispectral-demosaicing method that utilizes the outcomes of IGFPPI. The overall framework of the method is illustrated in Figure 3. We utilize the disparities between the estimated PPI and each channel, to generate the spectral-difference domain. We then perform directional interpolation of the unoccupied pixels in the spectral-difference domain. Finally, we add the interpolated image and the estimated PPI to obtain the final multispectral demosaicing result. In Figure 3, the masking block refers to the filtering of the raw image $I^{MSFA}$ to zero except for the corresponding channel position *c*.

The proposed directional-interpolation technique utilizes the interpolation order of the BTES method and weight calculation using the PPI. The BTES method first interpolates the center pixel in each step, resulting in four steps for a 4×4 MSFA, as shown in Figure 3. Here, the WC&WS block represents the weight calculation and weighted sum, where WC denotes the weight calculation and WS denotes the weighted sum. Let $\Delta_{s1}^{c}\left(i,j\right)$ represent the center pixel of the channel *c* requiring interpolation in the first step. The weight and weight-sum expressions in step 1 are given by (18) and (19), where s0 refers to step 0, s1 refers to step 1, and γ represents the weights.

The equations for step 2 are (20) and (21). Steps 3 and 4 are performed in the same manner as steps 1 and 2. Finally, the multispectral image is obtained by adding the estimated PPI to the spectral-difference image obtained through the order of BTES and directional interpolation, as follows:

(22) $$\begin{array}{c} {\hat{I}}^{c}={\hat{I}}_{PPI}+\Delta^{c}. \end{array}$$

## 4. Experiment Results

### 4.1. Metrics

To evaluate the quality of the demosaicing results, we used quantitative metrics, such as the PSNR, SSIM, and SAM.

The PSNR, which measures the logarithm of the average difference between the reference image and the estimated image, was calculated as follows:

(23) $$\begin{array}{cc} PSNR(x,\hat{x}) & =10\log_{10}\frac{{MAX}^{2}}{MSE(x,\hat{x})},\\ MSE(x,\hat{x}) & =\frac{{\left|\left|x-\hat{x}\right|\right|}^{2}}{WH}, \end{array}$$

where MAX represents the maximum value of the image, MSE represents the mean squared error between the reference image x and the estimated image $\hat{x}$, and *W* and *H* represent the width and height of the image, respectively.

The SSIM was used to evaluate the similarity between the reference image x and the estimated image $\hat{x}$. It was calculated using the following equation:

(24) $$\begin{array}{cc} SSIM(x,\hat{x}) & =\frac{\left(2\mu_{x}\mu_{\hat{x}}+c_{1}\right)\left(2\sigma_{x\hat{x}}+c_{2}\right)}{\left(\mu_{x}^{2}+\mu_{\hat{x}}^{2}+c_{1}\right)\left(\sigma_{x}^{2}+\sigma_{\hat{x}}^{2}+c_{2}\right)}, \end{array}$$

where $\mu_{x}$ and $\mu_{\hat{x}}$ represent the means of the image vectors x and $\hat{x}$, respectively. The standard deviations of x and $\hat{x}$ are represented by $\sigma_{x}$ and $\sigma_{\hat{x}}$, respectively. The covariance between x and $\hat{x}$ is represented by $\sigma_{x\hat{x}}$, and $c_{1}$ and $c_{2}$ are constants used to prevent the denominator from approaching zero.

The SAM is commonly used to evaluate multispectral images. It represents the average of the angles formed by the reference and estimated image vectors and is calculated using the following formula:

(25) $$\begin{array}{cc} SAM(x,\hat{x}) & =\cos^{-1}\left(\frac{x\cdot \hat{x}}{\left|\left|x\right|\right|\left|\left|\hat{x}\right|\right|}\right).\; \end{array}$$

For the PSNR and SSIM, larger values indicated better performance, and for the SAM, smaller values indicated better performance.

### 4.2. Dataset and Implementation Detail

In our experiments, we compared the proposed method to previously reported methods using the TokyoTech-31band (TT31) [23] and TokyoTech-59band (TT59) [24] datasets. The TT31 dataset included 35 multispectral images, each containing 31 spectral bands ranging from 420 to 720 nm. The TT59 dataset included 16 multispectral images with 59 spectral bands ranging from 420 to 1000 nm, with the bands spaced 10 nm apart. We excluded the popular CAVE [25] dataset from our experiments because it was used to train conventional deep-learning methods. To generate the synthetic dataset, we used IMEC’s “snapshot mosaic” multispectral camera sensor, i.e., XIMEA’s xiSpec [26]. We utilized the publicly available normalized transmittance of this camera [15] and the camera had the central spectral band $\lambda_{c}$ to be an MSFA of 4×4 arrays, consisting of 469, 480, 489, 499, 513, 524, 537, 551, 552, 566, 580, 590, 602, 613, 621, and 633 nm. The arrays were arranged in ascending order in Figure 1c. We used the normalized transmittance and D65 illuminant to obtain multispectral images for each band, in accordance with (1). The obtained images were then sampled using (2) to generate the raw MSFA images.

The pixel values of the synthesis datasets ranged from 0 to 1. The window size was set to h=7 and v=3 for horizontal guided filtering, h=3 and v=7 for vertical guided filtering, and h=5 and v=5 for the final guided upsampling. We also experimented with $10^{-4}$ for $\epsilon_{pixel}$, which determined the change in each pixel, and $10^{-3}$ for $\epsilon_{global}$, which determined the change in the entire image.

### 4.3. Results for Synthesis Dataset and Real-World Image

For a quantitative evaluation of the proposed method, we compared it to six other methods. The first conventional method (CM1) was the BTES method [13], which prioritized the interpolation of the empty center pixel in the spatial domain of each channel. Interpolation was performed using a weighted sum, and the weights were calculated using the reciprocal of the difference between neighboring pixels. The second conventional method (CM2) was a spectral-difference-(SD) method that employed weighted bilinear filtering in the spectral-difference domain [27]. The third conventional method (CM3) was an iterative-spectral-difference-(ItSD) method that used weighted bilinear filtering in the spectral-difference domain [28]. The CM2 method was applied repeatedly for each channel. The fourth conventional method (CM4) was an MLDI method similar to the BTES method of [14], except that the interpolation was performed in the spectral domain instead of the spatial domain. The fifth conventional method (CM5) was a PPID method that estimated the PPI of a guide image [15] and performed interpolation in the spectral-difference domain based on the PPI. The sixth conventional method (CM6) was a mosaic-convolution-attention-network-(MCAN) method, in which the mosaic pattern was erased by generating an end-to-end demosaicing network [16]. This deep-learning method was implemented using the code published online by the author.

**Figure 4:**
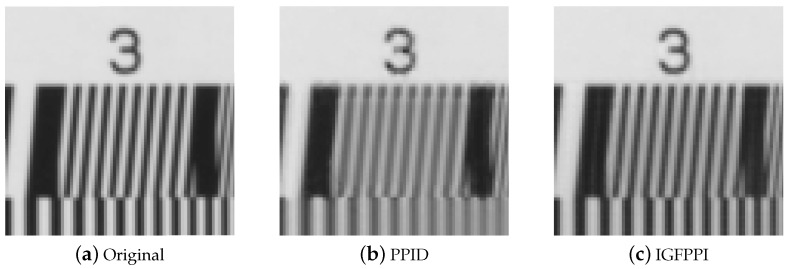
Experimental results for PPI estimation: (**a**) Original. (**b**) PPID. (**c**) IGFPPI.

Figure 4 shows the results of the estimated PPIs as a guide image. Figure 4a displays the average of the original multispectral cube, Figure 4b shows the estimated PPI of PPID [15], and Figure 4c shows the estimated PPI of the proposed IGFPPI. The estimated PPI of PPID is blurred and has low contrast. However, the proposed IGFPPI restored high-frequency components better than PPID, and the contrast is also close to the original.

The results for the PSNR, SSIM, and SAM of TT31 are presented in Table 1, Table 2 and Table 3, respectively. In the tables, a dark-gray background indicates the best score and a light-gray background indicates the second-best score. Of the 35 images in the TT31 dataset, the proposed method had the best PSNR for 19 images and the second-best PSNR for 16 images. Additionally, it had the best SSIM for 20 images, the second-best SSIM for 15 images, the best SAM for 18 images, and the second-best SAM for 17. The average PSNR, SSIM, and SAM values for the TT31 dataset indicated that the proposed method outperformed the other methods.

**Figure 5:**
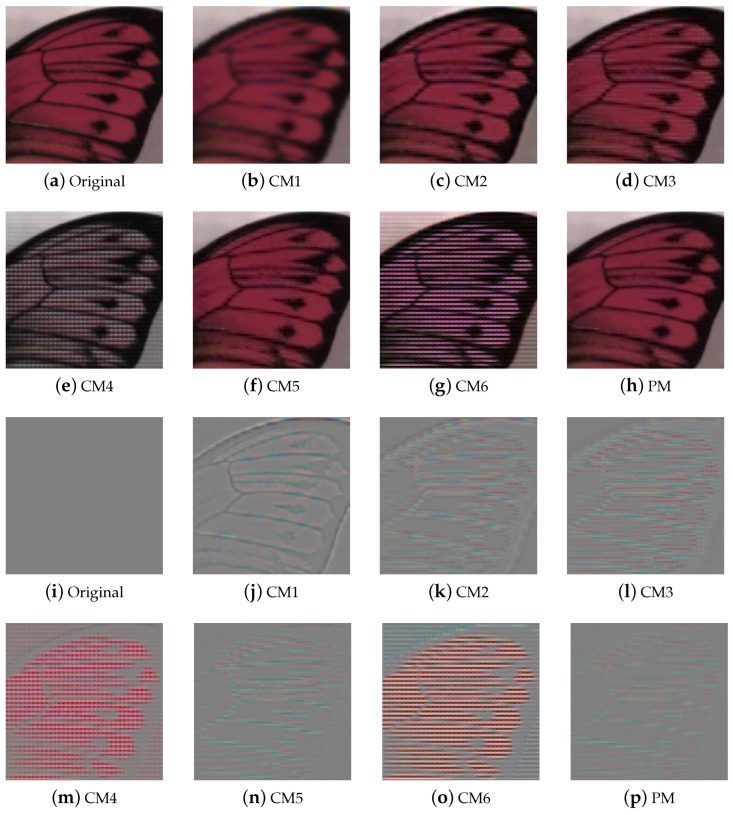
Experimental results for TT31: (**a**–**h**) Butterfly and (**i**–**p**) error maps of (**a**–**h**).

**Figure 6:**
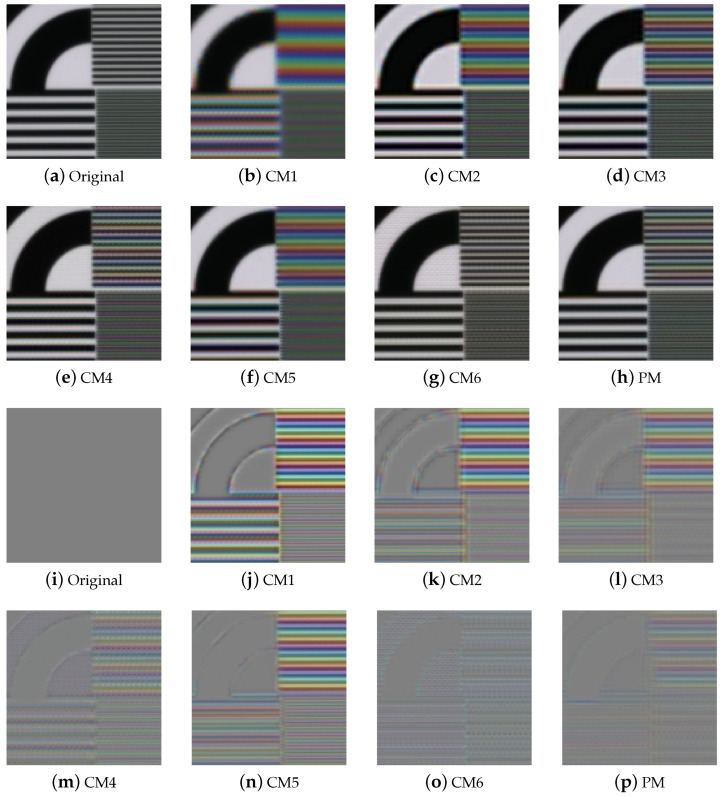
Experimental results for TT31: (**a**–**h**) ChartCZP and (**i**–**p**) error maps of (**a**–**h**).

Figure 5 and Figure 6 present the qualitative evaluation results for TT31, including those for the Butterfly and ChartCZP images, with the images cropped to highlight differences. We obtained red, green, and blue channels from the multispectral demosaicing image cube and represented them as the RGB images for qualitative evaluation. Figure 5a–h and Figure 6a–h show RGB images from which we extracted channel 16 for red, channel 6 for green, and channel 1 for blue from the multispectral image cube. Figure 5i–p and Figure 6i–p show the error maps of Figure 5a–h and Figure 6a–h. The results of CM1 show the blurriest image, and the results of CM2 and CM3 estimated high frequencies somewhat well, but artifacts can be seen. CM4 and CM6 nearly perfectly restored high frequencies in the resolution chart; however, the mosaic pattern was not entirely removed from the general color image. In CM6, demosaicing is performed using a network that erases the mosaic pattern for each channel. This method performs demosaicing on 16 channels of an MSFA; however, the arrangement is different from the paper of CM6. In the experimental results of this method, we can observe that only the evaluation metrics of chart images corresponding to monotone are of high score. This is because the mosaic pattern is easily erased in images where changes in all channels are constant, but the mosaic pattern is not erased in images where a large change occurs in a specific color. In general, the outcomes of CM5 and PM (referring to the proposed method) appeared to be similar. However, for images such as the resolution chart, PM exhibited superior high-frequency recovery and less color aliasing than CM5. Overall, the image produced by PM had fewer mosaic pattern artifacts and less color aliasing than those produced by the conventional methods.

For quantitative evaluation of the TT59 dataset, we computed the PSNR, SSIM, and SAM values, which are presented in Table 4, Table 5 and Table 6, respectively. Of the 16 images in the TT59 dataset, the proposed method had the best PSNR for 10 images, and the second-best PSNR for 4 images. Moreover, it had the best SSIM for 8 images, the second-best SSIM for 7 images, the best SAM for 12 images and the second-best SAM for 4 images. The average PSNR, SSIM, and SAM values for the TT59 dataset indicated that the proposed method achieved the best results.

The results for the TT59 dataset were similar to those for the TT31 dataset. In the gray areas, CM4 and CM6 effectively recovered the high frequencies. However, in the colored sections, MSFA pattern artifacts were introduced, resulting in grid-like artifacts. By comparison, CM5 and PM performed better overall, with PM recovering high frequencies better than CM5, as shown in the resolution chart.

**Figure 7:**
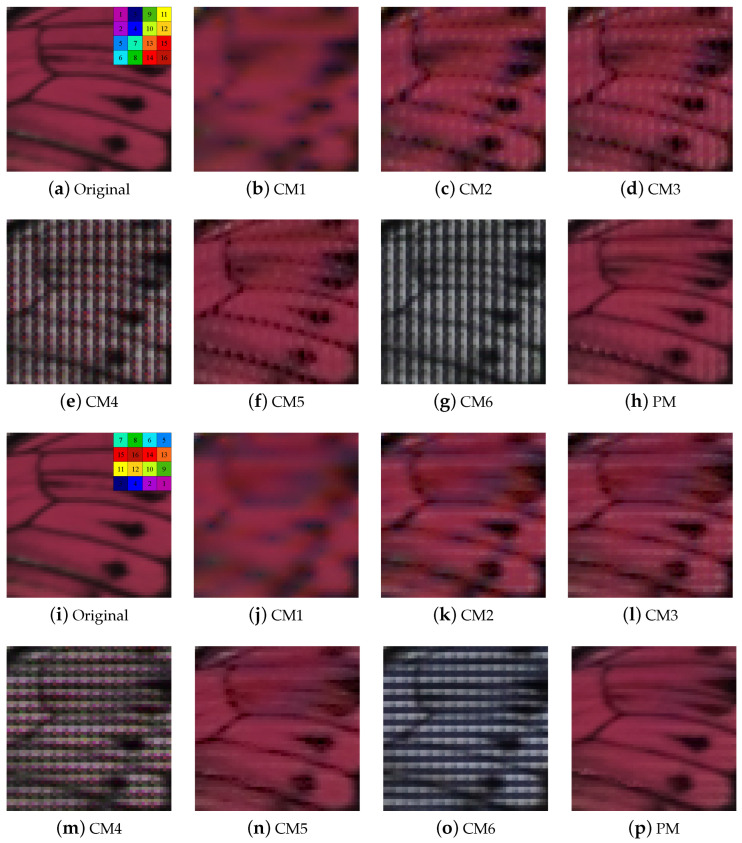
Experimental results for various MSFAs: (**a**–**h**) Demosaicing results for different arrangement MSFAs. (**i**–**p**) Demosaicing results for original MSFA.

Figure 7 shows the demosaicing results for different MSFA arrangements. Figure 7a–h shows the MSFAs in which adjacent spectra are grouped in a 2 × 2 shape. Figure 7i–p are the MSFAs of the original IMEC camera. The proposed method can be observed to be more robust and to have fewer artifacts than conventional methods. In particular, Figure 7c,d,f show grid artifacts where the black line of the butterfly is broken, whereas the proposed method shows reduced grid artifacts compared with other methods.

Table 7 presents a comparison of the execution times, with the desktop specifications of an Intel i7-11700k processor, 32 GB of memory, and an Nvidia RTX 3090 GPU. CM6 was tested using Pytorch, whereas the remaining methods were tested using MATLAB R2021a. To obtain the average execution times for all the datasets, we conducted timing measurements. We found that the method with the shortest execution time was CM1, followed by CM5, PM, CM4, CM2, CM6, and CM3.

**Figure 8:**
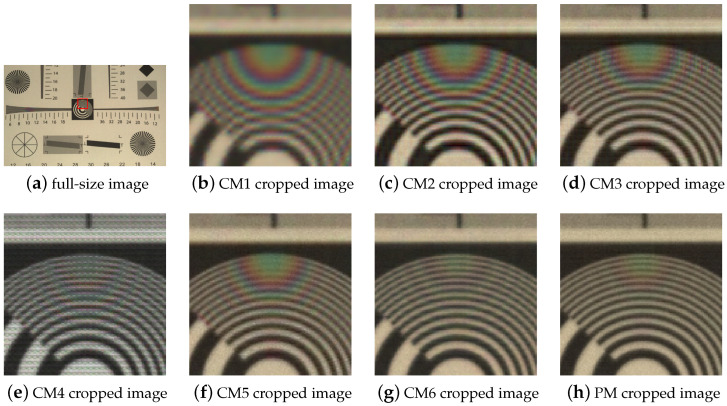
Real-world image.

In addition, as shown in Figure 8, the methods were tested on images captured using an IMEC camera. To qualitatively evaluate the multispectral image cube in the real world, we used the same method that was employed to evaluate the synthesis dataset. Channels 16, 6, and 1 of the multispectral image cube were extracted as the R, G, and B images, respectively, as shown in Figure 8. These results were similar to the experimental results obtained for the synthesis dataset. CM1, CM2, and CM3 exhibited blurred images and strong color aliasing, whereas CM4 exhibited MSFA pattern artifacts. Among the conventional methods, CM5 achieved the best results. CM6, which is a deep-learning method, performed well for the resolution chart. However, the proposed method exhibited better high-frequency recovery and less color aliasing.

## 5. Conclusions

We propose an IGFPPI method for PPI estimation and a directional-multispectral-demosaicing method using the estimated PPI obtained from IGFPPI. Guided filtering was used to estimate the PPI from the raw image of the MSFA, where a Gaussian filter was used to obtain the PPI of the low-frequency components, and horizontal and vertical guided filtering was used to estimate the high-frequency components. Using the estimated PPI, we performed directional interpolation in the spectral-difference domain to obtain the final demosaiced multispectral image.

In extensive experiments, among the methods tested, the proposed method achieved the best quantitative scores for the PSNR, SSIM, and SAM and exhibited the best restoration of high frequencies and the least color artifacts in a qualitative evaluation, with a reasonable computation time. The proposed method also achieved good results for real-world images. Furthermore, our proposed method can be adapted to perform multispectral demosaicing in the case of a periodic MSFA and when the spectral transmittance of the MSFA varies. In future research, we will focus on image-fusion demosaicing using both multispectral and color filter arrays.

## Figures and Tables

**Table 1:** PSNR(DB) Comparision for TT31.

	CM1	CM2	CM3	CM4	CM5	CM6	PM
Butterfly	32.28	36.07	37.50	25.17	39.95	19.94	41.85
Butterfly2	27.91	30.64	31.28	23.50	32.90	15.85	35.19
Butterfly3	34.05	38.40	40.53	29.96	43.84	26.39	43.73
Butterfly4	33.22	37.54	40.07	32.69	41.92	27.75	42.44
Butterfly5	33.26	37.92	41.37	36.16	43.64	31.96	43.89
Butterfly6	30.61	34.92	37.52	32.84	39.27	27.41	40.36
Butterfly7	33.89	38.59	41.60	33.72	43.45	27.56	43.51
Butterfly8	32.67	36.83	39.41	33.43	42.36	29.58	42.50
CD	38.69	39.31	37.28	24.32	40.54	24.81	38.77
Character	25.83	30.69	34.32	31.03	34.55	27.04	36.70
Chart24	34.65	38.43	40.50	28.01	41.71	23.38	42.65
ChartCZP	17.34	21.33	25.79	27.36	22.14	32.14	31.93
ChartDC	33.96	37.54	39.52	27.91	40.65	23.18	42.26
ChartRes	23.42	27.24	30.42	34.13	29.61	38.21	34.28
ChartSG	36.26	39.90	42.14	28.96	43.57	24.63	45.07
Cloth	26.95	31.89	34.77	31.41	35.93	26.84	35.77
Cloth2	31.64	35.16	37.44	27.75	39.58	22.37	38.68
Cloth3	32.07	34.97	36.34	29.09	37.30	21.64	37.76
Cloth4	29.69	34.07	36.58	29.59	37.77	24.44	39.57
Cloth5	34.31	36.11	37.00	21.13	37.57	21.41	39.62
Cloth6	38.50	41.25	41.95	32.21	43.47	25.90	45.54
Color	35.32	37.51	38.17	26.18	40.29	21.59	41.16
Colorchart	40.77	42.94	42.99	27.24	46.47	23.38	48.11
Doll	24.93	28.10	29.94	22.44	31.80	20.59	30.06
Fan	25.33	28.56	30.27	24.95	31.77	20.98	32.31
Fan2	26.83	30.91	32.96	26.17	34.80	20.73	34.58
Fan3	26.62	30.61	32.50	26.14	34.31	21.02	33.12
Flower	41.93	45.79	47.09	36.11	48.96	31.25	48.60
Flower2	44.00	46.51	46.27	30.83	48.02	27.34	48.42
Flower3	42.65	45.98	46.65	35.78	48.67	31.08	48.53
Party	29.49	32.62	33.45	26.10	34.64	21.75	33.39
Tape	30.24	32.62	33.84	21.17	35.19	16.36	33.83
Tape2	31.31	34.18	35.47	19.34	36.15	15.03	34.72
Tshirts	22.35	27.04	30.47	27.38	33.92	20.59	30.55
Tshirts2	25.21	29.15	32.21	28.54	34.79	21.92	32.91
Avg.	31.66	35.18	37.02	28.54	38.61	24.46	39.21

**Table 2:** SSIM Comparision for TT31.

	CM1	CM2	CM3	CM4	CM5	CM6	PM
Butterfly	0.924	0.959	0.961	0.656	0.978	0.441	0.984
Butterfly2	0.848	0.922	0.927	0.766	0.952	0.489	0.969
Butterfly3	0.961	0.973	0.975	0.851	0.988	0.775	0.990
Butterfly4	0.944	0.968	0.974	0.905	0.984	0.814	0.986
Butterfly5	0.958	0.980	0.986	0.928	0.991	0.851	0.991
Butterfly6	0.910	0.959	0.968	0.890	0.978	0.705	0.982
Butterfly7	0.960	0.980	0.984	0.892	0.990	0.727	0.990
Butterfly8	0.927	0.965	0.973	0.936	0.989	0.904	0.991
CD	0.983	0.974	0.957	0.823	0.984	0.823	0.976
Character	0.875	0.934	0.959	0.918	0.979	0.878	0.979
Chart24	0.956	0.970	0.975	0.784	0.986	0.715	0.987
ChartCZP	0.386	0.802	0.932	0.921	0.700	0.974	0.976
ChartDC	0.968	0.972	0.976	0.809	0.990	0.733	0.991
ChartRes	0.828	0.907	0.945	0.958	0.943	0.975	0.976
ChartSG	0.976	0.979	0.982	0.841	0.993	0.785	0.994
Cloth	0.775	0.929	0.953	0.920	0.965	0.842	0.961
Cloth2	0.843	0.929	0.948	0.670	0.962	0.398	0.973
Cloth3	0.847	0.924	0.937	0.736	0.947	0.386	0.959
Cloth4	0.732	0.922	0.949	0.798	0.956	0.579	0.972
Cloth5	0.831	0.911	0.922	0.444	0.929	0.519	0.955
Cloth6	0.924	0.967	0.972	0.829	0.980	0.545	0.986
Color	0.963	0.959	0.956	0.604	0.984	0.438	0.984
Colorchart	0.983	0.981	0.979	0.768	0.993	0.715	0.994
Doll	0.763	0.886	0.897	0.601	0.928	0.558	0.927
Fan	0.736	0.894	0.914	0.782	0.941	0.605	0.936
Fan2	0.857	0.934	0.943	0.749	0.962	0.568	0.953
Fan3	0.827	0.932	0.945	0.778	0.964	0.572	0.950
Flower	0.969	0.985	0.987	0.896	0.991	0.731	0.991
Flower2	0.975	0.985	0.983	0.754	0.988	0.592	0.988
Flower3	0.978	0.986	0.986	0.883	0.990	0.708	0.990
Party	0.93	0.956	0.957	0.778	0.972	0.620	0.971
Tape	0.879	0.934	0.941	0.593	0.956	0.447	0.949
Tape2	0.83	0.922	0.937	0.645	0.950	0.427	0.933
Tshirts	0.689	0.877	0.931	0.766	0.968	0.467	0.965
Tshirts2	0.676	0.876	0.935	0.765	0.963	0.434	0.967
Avg.	0.869	0.941	0.956	0.790	0.963	0.650	0.973

**Table 3:** SAM Comparison for TT31.

	CM1	CM2	CM3	CM4	CM5	CM6	PM
Butterfly	0.026	0.038	0.034	0.113	0.022	0.191	0.018
Butterfly2	0.059	0.087	0.078	0.136	0.049	0.290	0.045
Butterfly3	0.041	0.065	0.059	0.096	0.035	0.119	0.035
Butterfly4	0.072	0.097	0.084	0.114	0.059	0.128	0.058
Butterfly5	0.042	0.052	0.044	0.099	0.034	0.107	0.034
Butterfly6	0.040	0.050	0.039	0.064	0.027	0.067	0.027
Butterfly7	0.033	0.040	0.033	0.097	0.025	0.080	0.024
Butterfly8	0.076	0.117	0.096	0.092	0.055	0.090	0.051
CD	0.034	0.048	0.059	0.153	0.037	0.176	0.043
Character	0.084	0.155	0.118	0.088	0.061	0.095	0.057
Chart24	0.048	0.072	0.064	0.112	0.039	0.111	0.038
ChartCZP	0.198	0.274	0.141	0.125	0.149	0.058	0.059
ChartDC	0.041	0.066	0.060	0.101	0.035	0.106	0.035
ChartRes	0.050	0.077	0.049	0.029	0.034	0.019	0.019
ChartSG	0.051	0.084	0.075	0.108	0.045	0.119	0.043
Cloth	0.122	0.170	0.121	0.128	0.078	0.139	0.088
Cloth2	0.048	0.055	0.045	0.127	0.032	0.245	0.029
Cloth3	0.077	0.101	0.086	0.198	0.060	0.412	0.055
Cloth4	0.066	0.073	0.056	0.120	0.044	0.151	0.037
Cloth5	0.053	0.058	0.052	0.283	0.045	0.261	0.042
Cloth6	0.055	0.062	0.056	0.139	0.043	0.245	0.042
Color	0.029	0.039	0.038	0.168	0.024	0.172	0.024
Colorchart	0.042	0.068	0.069	0.134	0.039	0.139	0.037
Doll	0.086	0.110	0.102	0.270	0.073	0.246	0.078
Fan	0.074	0.100	0.078	0.101	0.047	0.179	0.044
Fan2	0.051	0.074	0.057	0.088	0.033	0.124	0.032
Fan3	0.061	0.079	0.062	0.119	0.038	0.157	0.040
Flower	0.064	0.083	0.079	0.188	0.057	0.419	0.061
Flower2	0.059	0.074	0.075	0.257	0.059	0.356	0.059
Flower3	0.070	0.089	0.089	0.223	0.068	0.385	0.073
Party	0.059	0.080	0.077	0.212	0.052	0.215	0.058
Tape	0.030	0.034	0.030	0.155	0.023	0.167	0.025
Tape2	0.052	0.074	0.064	0.155	0.041	0.307	0.044
Tshirts	0.099	0.171	0.131	0.115	0.051	0.188	0.062
Tshirts2	0.086	0.129	0.101	0.110	0.046	0.192	0.051
Avg.	0.062	0.087	0.072	0.138	0.047	0.184	0.045

**Table 4:** PSNR(DB) Comparision for TT59.

	CM1	CM2	CM3	CM4	CM5	CM6	PM
Butterfly	26.27	29.38	31.23	24.57	32.37	25.22	35.04
Butterfly2	30.78	34.19	36.58	32.33	38.21	34.7	41.89
Chart	21.75	25.40	28.37	34.09	27.58	41.31	32.54
Chart2	23.07	26.71	29.98	34.92	30.33	42.59	37.55
Chart3	20.84	24.08	26.87	32.94	25.60	41.91	30.43
Cloth	23.86	27.37	29.59	26.24	31.37	26.00	31.38
Cloth2	31.33	34.38	36.01	27.13	37.52	29.13	38.40
Cloth3	26.15	29.18	31.35	27.74	33.43	27.97	33.87
Doll	27.31	30.46	32.31	26.26	33.88	27.59	34.48
Doll2	30.10	33.75	35.92	30.27	37.66	31.82	38.20
Fan	29.24	33.16	34.88	25.87	36.36	27.85	36.53
Fan2	31.79	35.20	36.43	28.17	37.70	30.10	37.64
Fan3	26.62	30.18	32.03	26.52	33.45	27.89	33.02
Origami	27.00	30.19	31.95	25.90	33.70	28.14	35.48
Paint	25.05	28.61	30.8	22.84	32.13	24.68	31.10
Spray	24.80	27.95	30.29	27.71	30.67	28.18	33.95
Avg.	26.62	30.01	32.16	28.34	33.25	30.94	35.09

**Table 5:** SSIM Comparision for TT59.

	CM1	CM2	CM3	CM4	CM5	CM6	PM
Butterfly	0.851	0.929	0.944	0.826	0.964	0.873	0.972
Butterfly2	0.927	0.960	0.970	0.939	0.983	0.959	0.989
Chart	0.831	0.903	0.939	0.982	0.953	0.990	0.983
Chart2	0.851	0.912	0.943	0.983	0.968	0.990	0.989
Chart3	0.809	0.889	0.927	0.981	0.927	0.993	0.977
Cloth	0.699	0.888	0.918	0.624	0.943	0.639	0.941
Cloth2	0.841	0.932	0.945	0.625	0.958	0.784	0.971
Cloth3	0.801	0.898	0.921	0.784	0.954	0.796	0.959
Doll	0.845	0.910	0.923	0.792	0.955	0.826	0.963
Doll2	0.857	0.937	0.954	0.900	0.971	0.924	0.975
Fan	0.845	0.928	0.936	0.685	0.956	0.774	0.955
Fan2	0.876	0.937	0.940	0.750	0.957	0.808	0.952
Fan3	0.743	0.896	0.915	0.761	0.938	0.810	0.929
Origami	0.866	0.882	0.895	0.708	0.965	0.778	0.970
Paint	0.642	0.879	0.916	0.672	0.935	0.741	0.923
Spray	0.776	0.903	0.931	0.863	0.951	0.884	0.970
Avg.	0.816	0.911	0.932	0.805	0.955	0.848	0.964

**Table 6:** SAM Comparison for TT59.

	CM1	CM2	CM3	CM4	CM5	CM6	PM
Butterfly	0.047	0.067	0.054	0.084	0.034	0.067	0.028
Butterfly2	0.040	0.065	0.053	0.051	0.028	0.036	0.023
Chart	0.044	0.056	0.037	0.019	0.029	0.010	0.014
Chart2	0.037	0.053	0.036	0.016	0.023	0.009	0.011
Chart3	0.065	0.111	0.081	0.032	0.054	0.015	0.025
Cloth	0.121	0.158	0.127	0.229	0.084	0.213	0.077
Cloth2	0.066	0.079	0.066	0.244	0.049	0.161	0.042
Cloth3	0.128	0.190	0.169	0.181	0.100	0.163	0.088
Doll	0.131	0.187	0.173	0.195	0.110	0.160	0.099
Doll2	0.221	0.283	0.254	0.261	0.195	0.231	0.189
Fan	0.073	0.115	0.104	0.168	0.061	0.135	0.057
Fan2	0.092	0.136	0.122	0.169	0.084	0.129	0.078
Fan3	0.075	0.094	0.075	0.116	0.051	0.089	0.050
Origami	0.090	0.181	0.164	0.145	0.071	0.122	0.049
Paint	0.064	0.071	0.055	0.128	0.042	0.108	0.046
Spray	0.094	0.129	0.110	0.085	0.069	0.067	0.054
Avg.	0.087	0.123	0.105	0.133	0.068	0.107	0.058

**Table 7:** Computation Time of Methods.

	CM1	CM2	CM3	CM4	CM5	CM6	PM
Avg.	0.714 s	1.229 s	2.871 s	1.068 s	0.822 s	1.998 s	0.999 s

## Data Availability

Data are contained within the article.
